# Measuring eye states in functional MRI

**DOI:** 10.1186/s12868-016-0282-7

**Published:** 2016-07-13

**Authors:** Stefan Brodoehl, Otto W. Witte, Carsten M. Klingner

**Affiliations:** Hans Berger Department Neurology, Jena University Hospital, Erlanger Allee 101, 07747 Jena, Germany; Brain Imaging Center, Jena University Hospital, Jena, Germany

**Keywords:** Eye movements, Eye closure, Vision, EPI, fMRI

## Abstract

**Background:**

In many functional magnetic resonance imaging (fMRI) studies, experimental design often depends on the eye state (i.e., whether the participants had their eyes open or closed). Closed eyes during an fMRI is the general convention, particularly when patients are in a resting-state, but the eye state is difficult to verify. Although knowledge of the impact of the eye state on brain activity is steadily growing, only a few research groups have implemented standardized procedures to monitor eye movements and eye state. These procedures involve advanced methods that are costly (e.g., fMRI-compatible cameras) and often time-consuming (e.g., EEG/EOG).

**Results:**

We present a simple method that distinguishes open from closed eyes utilizing functional MR images alone. The utility of this method was demonstrated on fMRI data from 14 healthy subjects who had to open and close their eyes according to a predetermined protocol (3.0 T MRI scanner, EPI sequence with 3 × 3 × 3 mm voxels, TR 2.52 s).

**Conclusion:**

The method presented herein is capable of extracting the movement direction of the eyes. All described methods are applicable for pre- and post-normalized MR images and are freely available through a MATLAB toolbox.

## Background

The impact of eye closure on brain neurophysiology was first described by Hans Berger [1]. He reported altered EEG activity when participants closed their eyes. These changes in the EEG spectrum are often considered effects of visual perception/deprivation. More recent studies have demonstrated that the Berger effect is also present in complete darkness, which suggests its independence from the gathering of visual information [2]. Functional MRI findings further demonstrate that activity in multiple non-visual sensory areas is stimulated by eye closure [3–5], which suggests that eye state has a significant effect on multiple brain networks.

The impact of eye state was further underlined by studies of the resting-state network that demonstrated effective modulation of spontaneous brain activity according to the eye state in various systems [6–8]. Therefore, subjects are often instructed to lay still and keep their eyes closed, or “closed eyes” is used as a control or baseline condition in task-related fMRI studies [9]. However, most of these studies did not control the eye state and instead assumed that the subjects followed the instructions of the experimenters. An additional common problem in long-duration fMRI studies is the increased fatigue of participants, which may result in participants dozing off or even sleeping.

To observe vigilance and eye state, MRI-compatible EEG/EOGs are commonly used [10]. However, in addition to generating artifacts, prolonging the study time and increasing costs, our experience indicates that the use of EEG electrodes (including dermal cleaning solutions and conductive paste) is often poorly tolerated, particularly in studies involving patients. Moreover, because the EOG detects rapid eye movements and blinks based on a relative signal change, it is not well suited for differentiating “closed” and “open” eyes at a fixed points in time [11].

In the present study, we describe methods to extract the eye state (open vs. closed eyes) directly from recorded fMRI images. The reliability and effectiveness of these methods were tested in fMRI data from 14 subjects who were instructed to open and close their eyes following a predetermined protocol.

To enable our colleagues in the neuroscientific community, we presented this method within a MATLAB toolbox that is freely available (http://www.neuro.uniklinikum-jena.de/Forschung/AG+Neuroimaging.html or https://sourceforge.net/projects/eye-state-fmri/files) under the GNU public license for non-commercial use and open-source development.

## Methods

### Subjects

We examined 14 right-handed young adults [7 females; age range 21–27 years; mean age 23.2 ± 1.67 (mean ± standard deviation)]; see Table 1 for more details. No subjects reported any history of neurological or psychiatric disease. The investigations were performed according to the Declaration of Helsinki on Biomedical Studies Involving Human Subjects. The study was approved by the local ethics committee, and all subjects provided written informed consent according to the Declaration of Helsinki.

**Table 1:** Age and gender of subjects

	Age (years)	Gender	Handedness
#1	23	w	Right
#2	22	w	Right
#3	23	w	Right
#4	22	m	Right
#5	24	w	Right
#6	23	w	Right
#7	23	w	Right
#8	23	m	Right
#9	24	m	Right
#10	21	m	Right
#11	27	m	Right
#12	21	m	Right
#13	23	w	Right
#14	26	m	Right
Mean	23.2 ± 1.67	(7/14 male)	

All subjects were right-handed, as determined by the Edinburgh handedness inventory; any subject with neurologic diseases or peripheral dysesthesia were excluded

### MRI recordings

All experiments were performed using a 3.0-T MR scanner (Trio, Siemens, Erlangen, Germany) to obtain echo-planar T2*-weighted image volumes (EPI).

In the fMRI experiment, a block design was used. Starting with closed eyes, the subjects had to alternately open and close their eyes every 27 s (20 blocks each, total time of <25 min). Instructions to open and close the eyes were given verbally via headphones. In total, 600 EPI images (voxel size = 3 mm × 3 mm × 3 mm, TR = 2.52 s, TE = 35 ms; 40 transaxial slices, covering the entire cerebrum and cerebellum) were acquired.

EEG recordings: The first fMRI experiment with subject #1 (Table 1) was recorded with a simultaneous EEG (63 ring electrodes within an MRI-compatible cap (BRAINCAP-MR, BrainProducts) at a sampling rate of 5000 Hz, using a BrainProducts SyncBox to synchronize the EEG and fMRI data. For MR artifact correction, the BrainVisionAnalyzer 2.0 Software (BrainProducts) was used. The timing of probable eye opening and closing was defined via manual inspection by an experienced neurologist. These onsets were used to define the exact timing of the eye state vector (referred to as predetermined eye state in Figs. 3, 4, 5).

### Data analysis

Data analysis was performed on a PC using MATLAB (Mathworks, Natick, MA) and SPM12 software (Wellcome Department of Cognitive Neurology, London, UK, http://www.fil.ion.ucl.ac.uk/spm). The first three EPI volumes were discarded due to equilibration effects. All images were realigned to the first volume using six-parameter rigid-body transformations to correct for motion artifacts [12–14]. The images were co-registered with the corresponding anatomical (T1-weighted) images of the subject and normalized to the Montreal Neurological Institute (MNI) standard brain [15]. A standard smoothing kernel of 3 × 3 × 3 mm was applied to all images.

### Eye bulb analysis

A general description of the eyeball analysis is provided with more details to follow. (1) First, we generated a region of interest (ROI) that covered both eyes within the EPI images (covering 10–11 EPI slices). We performed a continuous max-flow algorithm to the ROI to segment the eye bulbs based on their intensity values [16]. (2) We then created a 3D shape of each extracted bulb and determined the greatest vector (diameter) in each 3D object. The vector was subsequently transformed into a norm (unity) vector. The vector in a 3D matrix consists of x/y/z values; we were primarily interested in the z dimension because it is correlated with the sagittal excursion of the eyes and because its change over time corresponds to eye opening and closing [17]. The angles of deviation from the x and z axis were calculated. (3) Additionally, we determined the mean MR intensity of each segmented bulb.

We analyzed the eye bulb on both realigned but not normalized images as well as realigned and normalized images (smoothed with a 3 × 3 × 3 Gaussian kernel).

#### Regions of interest (ROI) definition

For non-normalized EPI images, we defined the ROIs manually by using our provided toolbox. For normalized images, ROIs were defined using the following MNI coordinates: right [21 to 51] × [47 to 74] × [−50 to −26] and left [−48 to −18] × [45 to 74] × [−50 to −26] eye.

For image segmentation, we used an approach to the segmentation problem:

1 $$\min_{u} \left\langle {1 - u,C_{s} } \right\rangle + \left\langle {u,C_{t} } \right\rangle + \int\limits_{\varOmega } {\varpi \left( x \right)\left| { \nabla u} \right|dx}$$

as provided by Yuan et al. [16]. With this previously reported MATLAB implementation of a 2D/3D continuous max-flow method (CMF), we could automatically segment all ROI images.

**Fig. 1:**
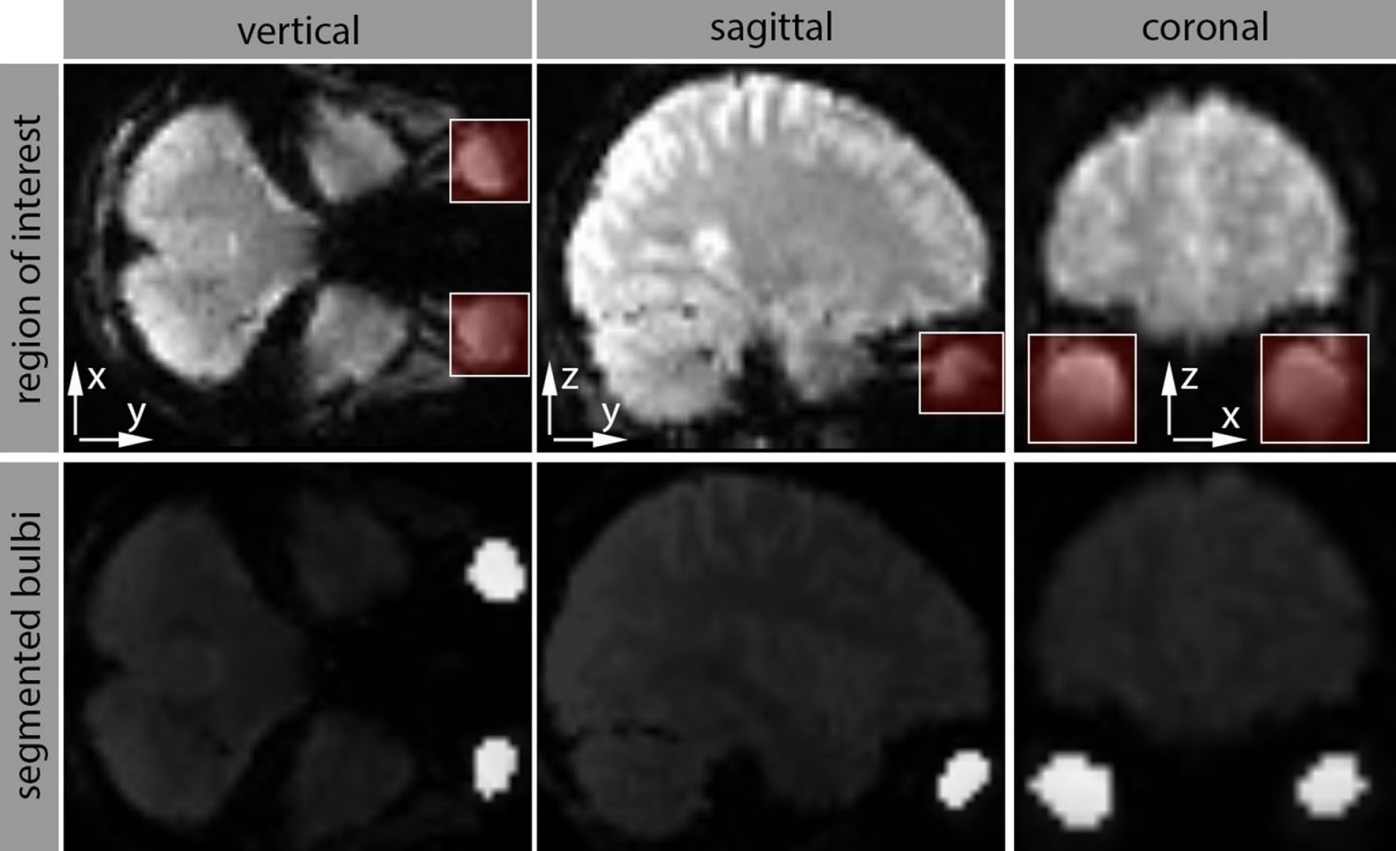
Regions of interest (ROIs) in normalized EPI images and segmentation. Example image: 53 × 63 × 52 (x/y/z dimension). Left ROI: [−48 to −18] × [45 to 74] × [−50 to −26] and right ROI [21 to 51] × [47 to 74] × [−50 to −26]. Defined ROIs are shown in the *upper row* in *white rectangles*. Segmented bulbs are shown in the *lower row* and highlighted in *white*

Figure 1 shows the results of the ROI definition and CMF segmentation for an exemplary EPI image.

#### 3D shape and maximum length vector

**Fig. 2:**
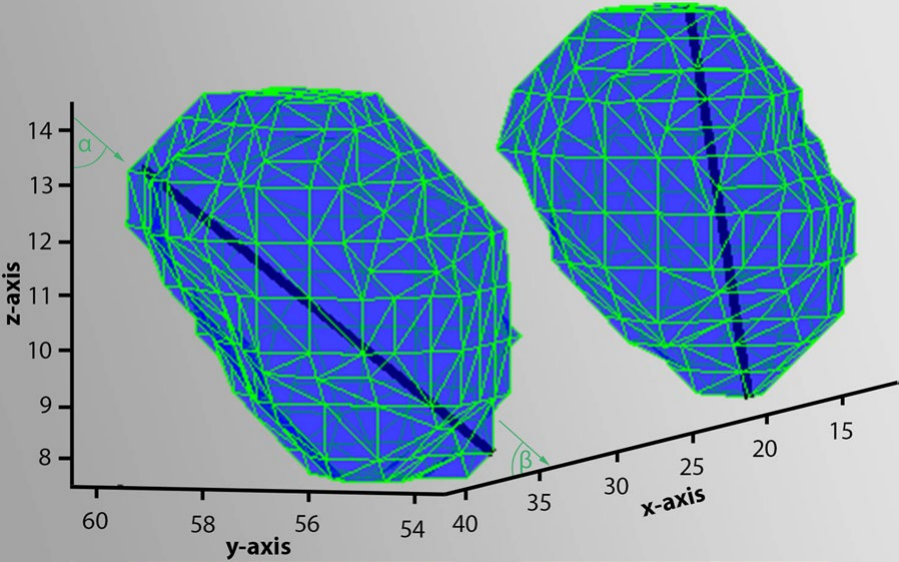
A 3D view of segmented bulbs in a subject with open eyes. x/y/z coordinates appear in NIFTI format; the *black thick line* in each bulb is the maximum length vector. The *horizontal* (β, between length vector and the *x*-axis) and *vertical* (α, between length vector and the *y*-axis) angles are shown

Using the segmented ROI data (Fig. 1), we created a 3D model using the MATLAB *isosurface* function. This creates vertices and shapes for a 3D model, as shown in Fig. 2. Using the Euclidean distance for each vertex, a maximum length vector, *u,* in each bulb was calculated. The unit vector was determined by $\hat{u} = \frac{u}{\parallel u \parallel}$. Because all operations are performed on a normalized space in a Cartesian coordinate system, the z dimension corresponds best to the up/down-movement of the eye bulb. Therefore, because eye closing and opening must lead to similar changes in vector orientation in both eyes, we determined the Pearson correlation coefficient between both eyes as a test condition.

To describe the direction of the length vector, we calculated the angle between the length vector and the x-axis (referred to as the horizontal angle) and the angle between the length vector and the z-axis (referred to as the vertical angle).

#### Mean intensity of segmented ROI

Tissue motion is known to reduce the MRI signal in echo planar imaging (EPI). This concept also applies to the vitreous of the eye [14, 18]. We therefore calculated the mean intensity for each time series image in the left, right and both eyes using the segmented ROI data.

To evaluate the quality of the eye position detection, the x/y/z dimensions of the length vector, angles and mean intensities were correlated with the predetermined eye position of the MRI block design.

## Results

### Bulb segmentation

A segmentation procedure of defined ROIs (manually in non-normalized and standardized in normalized EPI images) was applied. The manual inspection confirmed correct bulb configurations throughout the time series in all subjects.

The mean bulb sizes were 6910.9 ± 1736.3 mm^3^ (left eye) and 7162.9 ± 2404.2 mm^3^ (right eye) for not normalized and 7317.6 ± 961.6 mm^3^ (left eye) and 7162.9 ± 1081.8 mm^3^ (right eye) for normalized images. The correlation of bulb volume before and after normalization was 0.92 (Pearson correlation coefficient) for the left and right eye bulbs. Detailed results are outlined in Table 2.

**Table 2:** Eye bulb volumes of the segmented images before (left) and after (right) normalization

Subj.	Realigned only								Normalized							
	Size bulb (voxel)				Size (mm^3^)				Size bulb (voxel)				Size (mm^3^)			
	Left	SD	Right	SD	Left	SD	Right	SD	Left	SD	Right	SD	Left	SD	Right	SD
1	218.3	14.2	255.3	15.9	5893.7	382.6	6894.3	428.3	223.3	22.9	236.3	18.0	6029.6	618.0	6379.3	485.1
2	246.0	45.0	294.4	26.6	6642.6	1214.8	7948.6	719.3	256.1	31.3	244.0	23.1	6915.1	846.4	6587.1	622.7
3	153.7	16.4	172.7	21.6	4148.7	443.7	4664.2	584.3	213.5	75.7	227.7	95.4	5764.6	2044.5	6146.8	2574.9
4	315.6	47.7	382.0	33.2	8521.1	1288.8	10313.0	896.7	308.1	56.1	305.5	35.7	8319.4	1515.9	8248.1	962.7
5	372.8	31.6	377.2	43.7	10066.3	852.9	10183.1	1179.0	348.7	42.6	319.5	36.1	9414.2	1150.1	8627.2	975.9
6	289.8	37.8	334.6	36.7	7823.4	1020.3	9032.9	991.5	287.3	28.8	298.2	26.6	7758.1	777.6	8050.6	718.5
7	303.5	34.4	382.6	32.5	8195.1	928.9	10330.5	876.5	293.8	35.5	332.2	32.7	7932.1	958.6	8968.3	883.1
8	150.4	11.9	159.8	16.9	4059.5	320.8	4315.5	456.8	248.6	23.0	251.0	21.1	6711.3	621.2	6778.2	569.2
9	172.1	13.0	105.8	16.9	4646.3	352.1	2857.3	457.2	235.2	28.3	202.5	28.6	6349.3	762.8	5467.6	772.8
10	281.2	13.0	157.6	16.9	7592.6	352.1	4254.8	457.2	283.9	29.9	215.4	28.4	7665.7	808.3	5814.7	765.6
11	261.0	49.0	354.5	42.3	7047.4	1322.1	9572.1	1143.4	265.1	38.1	301.0	26.6	7157.6	1029.1	8126.9	717.6
12	327.9	30.6	279.2	22.9	8853.6	827.4	7539.0	618.7	293.0	38.1	254.5	17.4	7911.2	1030.0	6872.6	471.1
13	235.2	69.4	240.0	70.8	6351.7	1875.0	6479.9	1911.6	266.7	54.1	261.1	72.6	7201.2	1461.0	7050.0	1959.4
14	226.0	28.9	262.8	24.3	6101.6	780.6	7094.8	656.4	270.0	38.4	269.9	27.5	7289.9	1036.5	7287.8	743.0
MW	256 ± 64	269 ± 89	6911 ± 1736	7260 ± 2404	271 ± 34	265 ± 39	7318 ± 927	7163 ± 1043								

Images were recorded with a 3 × 3 × 3 mm resolution; therefore, the conversion factor from voxel volume to mm^3^ is 27. There was no significant difference between the bulb volumes before and after normalization (at p ≤ 0.05); however, volumes were correlated with 0.92 for the left and right eyes

### Eye positioning vector and angle

**Fig. 3:**
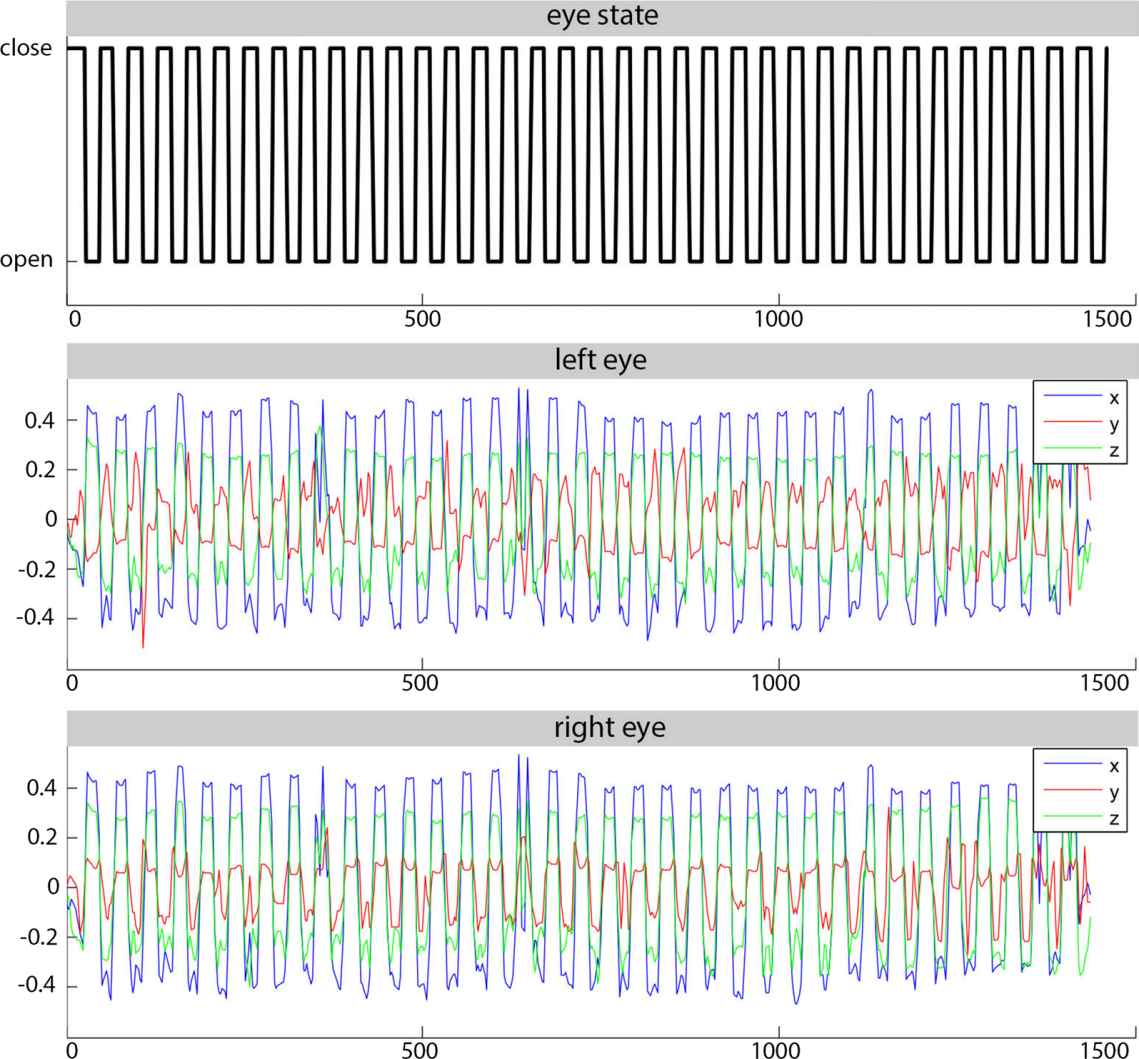
Norm vectors for the segmented eye bulbs (normalized images) in a subject. The *upper row* in *black* shows the predetermined eye state (1 closed, 0 opened). RB (right) and LB (left) are the x/y/z dimensions of the norm (unity) vector describing the absolute eye position at each given time point. The correlation coefficients for the x/y/z dimensions of the left and right eyes are as follows: x-dimension 0.978, y-dimension −0.636 and z-dimension 0.994. The correlations with predetermined eye states are x-dimension 0.919 (left) and 0.920 (right), y-dimension 0.704 (left) and −0.838 (right) and z-dimension 0.930 (left) and 0.938 (right). *X*-axis: time in seconds; *y*-axis of left/right eye: x/y/z dimensions of norm vector (in voxel)

For each segmented bulb, a length vector (defined by the maximum length diameter in the segmented ROI) was constructed. After converting the length vector to the norm vector, we analyzed the x/y/z dimension for each subject. Figure 3 shows an example of the results from one single subject (normalized images). To evaluate the quality of the method, we compared the x/y/z dimensions for the left and right eyes; for the subject shown in Fig. 3, there was a correlation of 0.978, −0.636 and 0.994 for the x-, y- and z-dimensions, respectively.

### Mean intensity

**Fig. 4:**
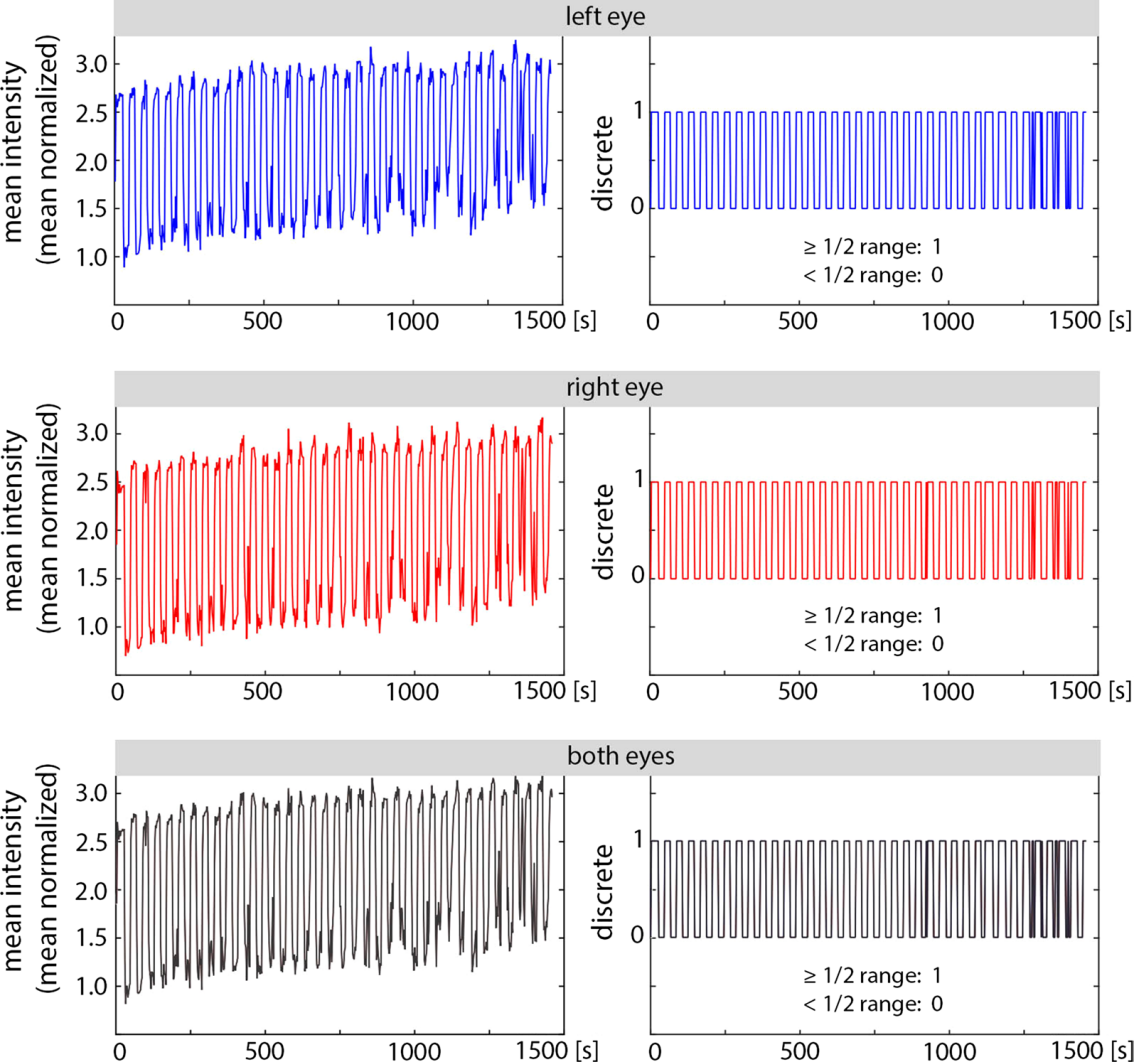
Mean intensity of the right, left and both bulbs of a single subject. Categorized values of 1 (values above ½ range) and 0 (below ½ range) are presented on the left. The correlation of the mean intensity of the left and right eyes was 0.971, and the correlation with the predefined eye state was left 0.91 (discrete 0.98), right 0.92 (discrete 0.99), both eyes 0.91 (discrete 0.99)

Furthermore, we calculated the mean MRI signal intensity of each eyeball. Figure 4 shows the results for the same subject depicted in Fig. 3. The correlation of the intensity values of the left and right eyes was 0.971. We categorized the signal intensity as a value of 1 (intensity above ½ range) or 0 (below ½ range). The categorized values were highly correlated with the predefined eye state (Pearson correlation coefficient above 0.98).

### Group results

**Fig. 5:**
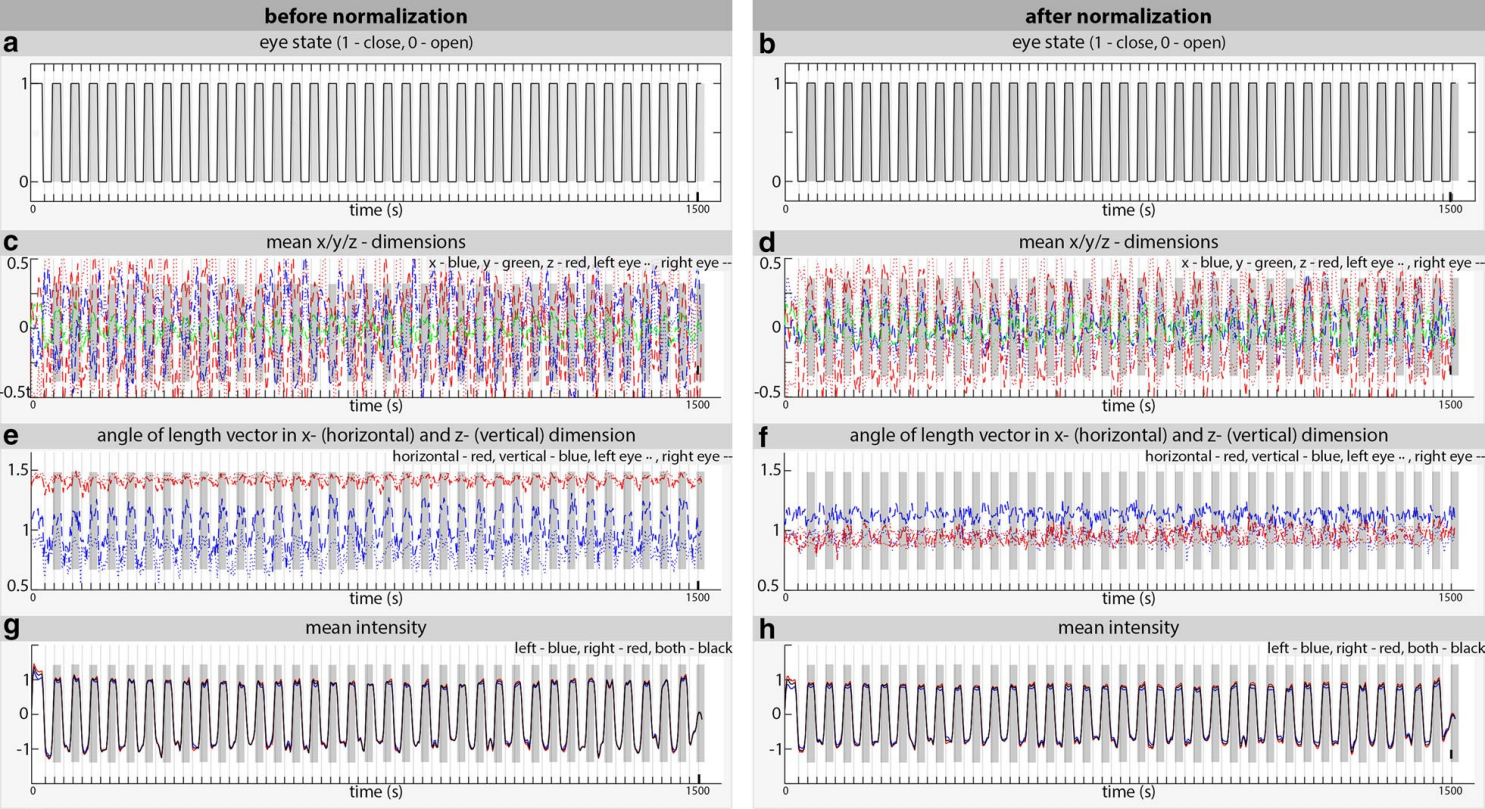
Summarized results for n = 14 subjects. The results before image normalization are presented on the left (**a**, **c**, **e**, **g**); the results after image normalization are presented on the right (**b**, **d**, **f**, **h**). *Grey bars* indicate eyes closed; for better visualization, these *bars* are overlaid on all subfigures below. **a**/**b** the predetermined eye state as verbally instructed during MRI recording. **c**/**d** x/y/z dimensions of the normalized length vector (unity vector with length of 1 voxel) of each bulb. Correlations between the left and right bulb: (before normalization) x-dimension 0.70, y-dimension −0.77, z-dimension 0.82; (after normalization) x-dimension 0.43, y-dimension 0.89, z-dimension 0.81. Correlation with the predetermined eye state: (before normalization) x/left 0.76, x/right 0.82, y/left −0.80, y/right 0.78, z/left 0.79, z/right 0.85; (after normalization) x/left 0.74, x/right 0.50, y/left 0.90, y/right 0.87, z/left 0.82, z/right 0.75. **e**/**f** Angle of the length vector in the x- and z-dimension. The angles were calculated in radians. Correlations between the left and right bulb: (before normalization) *horizontal* 0.77, *vertical* 0.88; (after normalization) *horizontal* 0.67, *vertical* 0.55. Correlations with the predetermined eye state: (before normalization) *horizontal left* 0.72, right 0.75, *vertical left* 0.86, right 0.90; (after normalization) *horizontal left* 0.79, right 0.74, *vertical left* 0.80, right 0.69. **g**/**h** Mean intensity of the segmented eye bulb (low-/high-pass filtered 0.1–0.01 Hz). Correlations between the left and right bulb (before normalization) 0.998; (after normalization) 0.998. Correlations with the predetermined eye state: (before normalization) left 0.947, right 0.946, both eyes 0.947; (after normalization) left 0.950, right 0.951, both eyes 0.951

Summarized results for all 14 participants are depicted in Fig. 5; the x-, y- and z-dimensions of the normed length vector were correlated with the exact eye state (before normalization: x_left_ 0.76, x_right_ 0.82, y_left_ −0.80, y_right_ 0.78, z_left_ 0.79, z_right_ 0.85; after normalization: x_left_ 0.74, x_right_ 0.50, y_left_ 0.90, y_right_ 0.87, z_left_ 0.82, z_right_ 0.75). There was no significant difference when applying the procedure either before or after image normalization (as measured by a two-sample *t* test with a threshold of p ≤ 0.05) (Fig. 5c, d). By calculating the angle between the x-axis and z-axis, we defined a horizontal (between length vector and x-axis [1 0 0]) and a vertical (between length vector and z-axis [0 0 1]) angle (see Fig. 2 for illustration). Before normalization, the correlations between the angles and the known eye states were 0.72 for the horizontal left, 0.75 for the horizontal right, 0.86 for the vertical left and 0.90 for the vertical right. After normalization, the correlation values were 0.79 for the horizontal left, 0.74 for the horizontal right, 0.80 for the vertical left and 0.69 for the vertical right. The highest correlations were found for the vertical angles. However, the correlation in pre-normalized images was superior to that found in the normalized images (correlation coefficient of 0.86 vs. 0.80 for the left eye and 0.90 vs. 0.69 for the right eye) (Fig. 5e, f).

The highest correlation with the predetermined eye state was found for the mean intensity of the segmented eye bulb: the values of 0.947 before and 0.951 after normalization (in the left and right eye) indicated that the normalization procedure was negligible. In Fig. 5g, h, the filtered mean intensities of all the images are shown. To adjust for the steady change in MRI signals, we applied a low-/high-pass filter (0.1–0.01 Hz) and normalized the data with zero to mean, 1 to max and −1 to min values. To exclude a potential systematic error, two different regions of interest with the same cluster size were defined (one within the right ventricular system and one within the left sided white matter); in these areas, the correlation coefficients with eye state were below 0.2 in all images.

In addition to demonstrating that eye state can be detected with a functional MRI signal, we intended to retrospectively determine whether participants had their eyes open or closed. Therefore, we randomly selected 10 % of the images from each series/subject and excluded the data from a second analysis. Because the results were most promising (highest correlation with eye state) for the mean intensity and the horizontal angle in the first analysis, we focused on these two parameters. By analyzing the last 90 % of the images, we obtained the distribution (range) of the mean intensity and angles. The top 10 % lowest and highest values (after low-/high-pass filtering) were used to categorize the eyes as closed (high mean intensity and great angle) or open (low mean intensity and low angle) (Fig. 5e–h). Subsequently, these individual criteria were used in the excluded 10 % images, which were automatically labeled as “closed” or “opened”. We calculated a mean congruency with the predetermined eye state of 95 % (range 92–99 %) for the images obtained before and 93 % (range 90–96 %) for the images obtained after normalization in all participants.

## Discussion

In the present study, we demonstrated a method to determine whether human subjects had their eyes open or closed based solely on recorded functional MR images. The key features of this method are the automated segmentation of the eye bulb, the analysis of distinct changes of the spatial dimensions and the MR intensity within the eye bulb.

A critical initial step in this method is the segmentation of the eye bulb. The exact segmentation is difficult because of the relatively low spatial resolution of the fMRI data (e.g., 3 × 3 × 3 mm) and the resulting partial volume effects on the edge of the eyeball. If the segmentation algorithm is too spatially restricted, then the border area might be falsely excluded. This results in a compromised identification of the oval shape of the eye bulb that causes a loss of signal in the border area of the eye bulb where eye movements exhibit the highest velocity and the strongest signal changes. On the other hand, if the segmentation algorithm is less restrictive and includes too much volume, then a lower performance may result because the portion of the vitreous within the whole volume is reduced. Therefore, a precise detection of the correct borders of the eye bulb is necessary. Although the current method demonstrated that the volume of the segmented eyeball fits well to the mean size of an adult eyeball of 7180 mm^3^ [19], further improvements in the segmentation of the eye bulb should improve the accuracy of this method in determining eye state.

Another discussion point is the reduction of the geometric 3D structure of the eye to a single value. We decided to focus on the excursion of a representative length vector within the eye. Because the eye is an oval shape, this length vector changes when the eye moves. The most significant correlation to eye state was found for the z-dimension, which corresponds to upward/downward movements of the eye and has larger values when the eyes are closed (upward) and smaller values when the eyes are open (downward). Accordingly, we revealed a high correlation of larger vertical angles in closed eyes. This is well in line with the physiology of the eye, where eye closure leads to an upward eye position in most humans, a phenomenon that was described by Charles Bell (1825). However, Bell came to this conclusion based on two observations: (1) in a peripheral lesion of the facial nerve or a mechanical impairment of the eyelid, closure was associated with elevation of the uncovered eye (Bell’s palsy), and (2) when putting the finger over a closed eye and blinking with the other eye, he felt the cornea under the lid roll upward. Using photographic techniques and electrooculographic (EOG) recordings a century later, a mean eye elevation of 20-60° was estimated [20–22]. The extent of eye elevation during eye closure is highly variable and ranges from a strong upward deviation to an undetectable or even downward deviation in some humans [23]. In our study, the mean elevation of both eyes within all subjects was approximately 28° (0.49 in radian measure) and thus fits well within the range of physiological specifications.

Finally, we compared the MRI signal of the segmented eye bulb in closed and open eyes. Considering that the MRI signal is lower in moving tissue [24] and eye movements are reduced in closed eyes [17, 25], we found, as expected, a reduced MRI signal associated with open eyes in the segmented eye bulb. A similar methodology was presented by Beauchamp [14], who reported greater MRI signal variance when subjects voluntarily moved their eyes. In contrast to the study by Beauchamp, the determination of the eye state with the current method is far less dependent on the TR of the measurement.

The accuracy of the current method to discriminate closed from open eyes was tested by using a jackknife algorithm, which demonstrated a 93–95 % rate of correct labeling of the eye state.

Correctly labeling the eye state 100 % of the time is unattainable due to multiple factors, the first of which is signal quality. The appearance of MR artifacts in EPI measurements, particularly in the frontal brain regions, can alter the extracted MRI signal of the eye bulb. Because the algorithm relies primarily on changes in the MRI signal, such artifacts can negatively impact the accuracy of the current method. Next, smoothing the data with a Gaussian kernel is necessary for improving the signal to noise ratio by removing signal artifacts and increasing the reproducibility of the segmentation algorithm. However, by smoothing the data, several drawbacks arise, including blurred edges and reduced spatial resolution. By varying kernel settings (none and resolution from 3 to 9 mm) in our study, we found a compromise between the accuracy of the segmentation procedure and the spatial accuracy for a kernel with 3 × 3 × 3 mm. Furthermore, due to the relatively slow TR time of 2–3 s, it is possible that eye opening can be interrupted by eye blinks. If an eye blink occurs during slice acquisition, the MRI signal at this time is most likely different from the eye open condition. However, to estimate the effect of blinking on the acquired MRI signal, the duration of the blink and the eyeball excursion must be considered. There is consensus regarding the duration of an eye blink of approximately 200–300 ms [26]. However, how the eyeball moves when blinking is quite controversial. Bell initially proposed an upward rotation of the eyes during blinking. More recent findings, however, suggest that intended and reflexive blinking are associated with a fast downward deviation. Only after 2 s of eye closure does a tonic upward deviation appear, with a movement of no more than 20–30°/s [27]. Consequently, blinking should affect a single EPI image but should not affect the previous and succeeding images. To compensate for the detection of false eye movements, we applied a low-pass filter to remove blinking artifacts. We did not measure the actual eye state with another objective method, such as EOG or video recording, in each subject. Therefore, we have to consider that some subjects may not have followed the verbal instructions to open and close their eyes accurately.

Furthermore, we considered another promising approach to further improve the prediction of eye state and evaluated the absolute position of the eyeball in the orbit. Eye closure is associated with a secondary eyeball retraction of 1–2 mm into the orbit, which is most likely due to the contraction of the extra orbital muscles [26, 28]. Due to the spatial resolution of 2–3 mm, which is normally applied in EPI images, and the smoothing to a 3 × 3 × 3 Gaussian kernel that was performed in our study, detection of this movement was not available with our method.

Our methodological approach was successful for pre- and post-normalized images. The best results, however, were achieved with pre-normalized images. It appears that the normalizing process warps the ovoid eye toward a more spherical structure, which results in a less accurate determination of the length and diameter and less accurate eye movement detection. However, the use of normalized images reduces the effort required to define the region of the eye balls (ROI) and can be employed for fully automated processing. We therefore suggest that both approaches be evaluated according to the intended experimental purpose.

Acquisition of EEG/EOG is the most adequate method for determining the exact onset of eye opening or closure as well as eye blinks. This process works by noting the muscular artifacts in the EEG/EOG signal and evaluating changes in the frequency spectrum (i.e., deceleration upon eye closure). However, the detection of the eyes as open or closed at a given time period is often hindered by the lack of a reference point. Moreover, the EOG cannot reliably detect the absolute eye position during lid movements or when the eyes are closed [11]. Other electrophysiological methods that are commonly used to identify absolute eye position—such as the double magnetic induction method (DMI) or magnetic coils—are not applicable in the MR environment because of their electric nature [29, 30]. In recent MR studies, new approaches to measure eye movement and position using an infrared pupil tracking system [14] or a search-coil eye tracker [31] have been introduced. However, the major advantage of our proposed method is that it is cost neutral and is particularly well suited to determining the eye state retrospectively from fMRI data alone.

To our knowledge, this is the first study to demonstrate a successful determination of the eye state from common functional MR images. To reproduce and improve our approach, we provide all of the described methods in a MATLAB toolbox for free download.

## Conclusion

We present a retrospective method to determine whether participants had their eyes open or closed based on functional MR images. We propose that this method is particularly useful for retrospective analyses or meta-analyses of fMRI data in the absence of EEG/EOG data when the knowledge of eye state is critical; this method may also permit insight into already published data.

All of our methods are outlined in the freely available MATLAB toolbox (http://www.neuro.uniklinikum-jena.de/Forschung/AG+Neuroimaging.html or https://sourceforge.net/projects/eye-state-fmri/files) under the GNU public license for non-commercial use and open-source development.

## Abbreviations

CMF: continuous max-flow method
EEG: electroencephalogram
EOG: electrooculography
EPI: echo planar imaging
fMRI: functional magnetic resonance imaging
MR: magnet resonance
ROI: region of interest
TR: repetition time
TE: echo time
